# Liraglutide Increases FGF-21 Activity and Insulin Sensitivity in High Fat Diet and Adiponectin Knockdown Induced Insulin Resistance

**DOI:** 10.1371/journal.pone.0048392

**Published:** 2012-11-12

**Authors:** Mengliu Yang, Lili Zhang, Chong Wang, Hua Liu, Guenther Boden, Gangyi Yang, Ling Li

**Affiliations:** 1 Key Laboratory of Diagnostic Medicine (Ministry of Education) and Department of Clinical Biochemistry, College of Laboratory Medicine, Chongqing Medical University, Chongqing, China; 2 Department of Pediatrics, University of Mississippi Medical Center, Jackson, Mississippi, United States of America; 3 The Division of Endocrinology/Diabetes/Metabolism and the Clinical Research Center, Temple University School of Medicine, Philadelphia, Pennsylvania, United States of America; Visva Bharati University, India

## Abstract

**Background:**

Liraglutide is a glucagon-like peptide-1 analogue that stimulates insulin secretion and improves β-cell function. However, it is not clear whether liraglutide achieves its glucose lowering effect only by its known effects or whether other as yet unknown mechanisms are involved. The aim of this study was to examine the effects of liraglutide on Fibroblast growth factor-21 (FGF-21) activity in High-fat diet (HFD) fed ApoE^−/−^ mice with adiponectin (Acrp30) knockdown.

**Method:**

HFD-fed ApoE**^−/−^** mice were treated with adenovirus vectors expressing *shAcrp30* to produce insulin resistance. Hyperinsulinemic-euglycemic clamp studies were performed to evaluate insulin sensitivity of the mouse model. QRT-PCR and Western blot were used to measure the mRNA and protein expression of the target genes.

**Results:**

The combination of HFD, ApoE deficiency, and hypoadiponectinemia resulted in an additive effect on insulin resistance. FGF-21 mRNA expressions in both liver and adipose tissues were significantly increased while FGF-21 receptor 1 (FGFR-1) and β-Klotho mRNA levels in adipose tissue, as well as FGFR-1-3 and β-Klotho mRNA levels in liver were significantly decreased in this model. Liraglutide treatment markedly improved insulin resistance and increased FGF-21 expression in liver and FGFR-3 in adipose tissue, restored β-Klotho mRNA expression in adipose tissue as well as FGFR-1-3, β-Klotho levels and phosphorylation of FGFR1 up to the levels observed in control mice in liver. Liraglutide treatment also further increased FGF-21 proteins in liver and plasma. In addition, as shown by hyperinsulinemic-euglycemic clamp, liraglutide treatment also markedly improved glucose metabolism and insulin sensitivity in these animals.

**Conclusion:**

These findings demonstrate an additive effect of HFD, ApoE deficiency, and adiponectin knockdown on insulin resistance and unveil that the regulation of glucose metabolism and insulin sensitivity by liraglutide may be partly mediated via increased FGF-21 and its receptors action.

## Introduction

Glucagon-like peptide (GLP-1) is a gut hormone secreted in response to ingestion of carbohydrates, lipids, and mixed meals from the L-cells located in the distal jejunum, ileum and colon/rectum [1], [2]. It acts as an incretin hormone by potentiating glucose-stimulated insulin release. GLP-1’s effect on beta cells has been documented in both animal and human studies [3]–[7]. Furthermore, GLP-1 has been shown to suppress hepatic glucose output [8], [9] and to decrease the rate of gastric emptying in patients with Type 2 diabetes mellitus (T2DM) [10]. However, the use of GLP-1 in diabetic patients is limited by its short t1/2. Liraglutide is a GLP-1 analogue with 97% sequence identity to human GLP-1 [11], [12]. It has been approved by the FDA in January 2010 for treatment of hyperglycemia in patients with T2DM. In previous studies, we have demonstrated that exenatide, a GLP-1 receptor agonist, could increase adiponectin (Acrp30) expression in high fat diet (HFD) induced insulin resistant rats and in rats fed a normal chow diet [13]. However, the mechanisms by which liraglutide improves insulin resistance are still not completely understood and may involve effects on release of certain cytokines.

Fibroblast growth factor-21 (FGF-21) is a regulator of insulin action on glucose and lipid metabolism. It is produced predominantly by the liver and, to a lesser extent, by adipose tissue. The biological actions of FGF-21 are elicited upon its β-klotho facilitated binding to FGF receptors (FGFR), leading to rapid dimerization and autophosphorylation of the FGFRs.

Kharitonenkov et al. have reported that the administration of FGF-21 to obese leptin-deficient *ob/ob* mice, to leptin receptor-deficient *db/db* mice or to obese ZDF rats lowered blood glucose, triglyceride, and insulin levels and improved glucose clearance during an oral glucose tolerance test [14]. In previous studies, we have shown that plasma FGF-21 levels are elevated in patients with T2DM and in diabetic patients with ketosis [15], [16], and are decreased in response to treatment with rosiglitazone [17]. This suggested that there may be FGF-21 resistance in these patients and that elevation of FGF-21 might be a compensatory mechanism to improve impaired insulin action. Together, these observations also suggested that FGF-21 may be a therapeutic target for treatment of diabetes associated with insulin resistance. However, whether liraglutide affects FGF-21 activity is unknown.

Abnormalities in lipid and especially free fatty acid (FFA) levels frequently precede the development of T2DM [18], [19]. ApoE^−/−^ mice develop severe hypercholesterolemia and spontaneous atherosclerosis, on a normal chow diet [20], [21] and are widely used as a model of insulin resistance when fed by a HFD [22]–[24].

Adiponectin is an adipocyte-derived hormone with important roles in the regulation of insulin sensitivity. Adiponectin knock-out mice on a HFD have high plasma FFA and severe insulin resistance [25]. Plasma adiponectin levels are reduced in obese humans and correlated inversely with insulin resistance. Therefore, obese patients with T2DM have hypoadiponectinemia and are insulin resistant. However, whether the combination of HFD, ApoE deficiency, and hypoadiponectinemia has an additive effect on insulin resistance is unknown.

In this study, by using the ApoE^−/−^ mice, we hypothesized that 1) the combination of HFD, hyperlipidemia, and adiponectin knockdown may cause more severe insulin resistance and 2) that the insulin resistance ameliorating effects of liraglutide was, at least in part, due to increased FGF-21 activity.

## Materials and Methods

### Construction and Purification of Adenoviruses Expressing Short Hairpin RNA (shRNA) Against Adiponectin

In order to construct adenoviral vectors expressing shRNAs against *Acrp30*, we designed 3 oligonucleotides and complementary strands to target specifically mouse *Acrp30*. The AdEasy™ XL system (Stratagene) was used for the shRNA construction. Briefly, the top and bottom oligonucleotides were annealed and ligated into the pSilencer1.0-U6 vector (Stratagene), and the sequence was confirmed. To evaluate the potency of these shRNAs in vitro, adiponectin shRNAs expression plasmids were transfected into 3T3-L1 adipocytes. Cells were harvested 48 hours later for testing of knockdown efficiency. In order to generate the recombinant adenovirus vectors expressing shRNAs for adiponectin(Ad-sh*Acrp30*), selected pSilencer1.0- U6-shRNA plasmids were recombined into the Gateway-based pAd- AdEasy™ XL vector (Stratagene), according to the manufacturer’s instructions. As a negative control, a recombinant adenovirus vector expressing a shRNA directed against Green Fluorescent Protein (Ad-*shGFP*) was generated. Amplification of recombinant adenovirus was performed according to the manufacturer’s instructions (Stratagene) using HEK 293A cells. Large-scale amplification and purification of recombinant adenoviruses were performed using the ViraBind Adenovirus Purification Kit according to the manufacturer’s instructions (Cell Biolabs Inc.).

### Animals

Male ApoE^−/−^ mice were purchased from The Experimental Animal Center of Beijing University of Medical Sciences (Beijing, China) at 3 weeks of age, acclimated for a week, and fed a regular chow or a HFD (33% carbohydrate, 13% protein, and 54% fat**)** for 16 weeks. Ad-*shGFP* or Ad-*shAcrp30* was injected by tail vein at the end of both the 14th and 15th week of HFD feeding. For the dose-response experiment, Ad-*shAcrp30* (1×10^9^ pfu in 100 µl of PBS) was administered by injection into the tail vein of mice at 50, 100, 150 or 200 µl (three animals per treatment group, (Fig. S1A). For determining time course of effects of Ad-*shAcrp30* (100 µl, 1×10^9^PFU), adipose tissues were collected 1 day prior to and 3, 5, 7 days post injection (three animals per group, Fig. S1B) and immediately deep-frozen in liquid nitrogen for mRNA analysis. Liraglutide (1 mg/kg) or saline was given intraperitoneally twice daily for 8 weeks (from 9th to 16th week of HFD feeding). ApoE^−/−^ mice were divided into following treatment groups: regular chow (Control group), HFD (HF group), HFD plus Ad-*shGF*P (Ad-*shGFP* group), HFD plus Ad-*shAcrp30* (Ad-*shAcrp30* group), HFD plus Ad-shAcrp30 with liraglutide treatment at a dose of 1 mg/kg twice daily (Liraglutide group), and HFD plus Ad-*shAcrp30* with saline injection (Saline group). Mice were housed in individual cages in a temperature- and light-controlled (12-hour light/12-hour dark cycle) facility. Prior to the hyperinsulinemic-euglycemic clamp studies, intracarotid arterial and intrajugular venous catheters were placed for infusions and blood sampling, respectively. The intrajugular venous catheters were also used for intravenous glucose tolerance tests. Animals were allowed to recover for 3 days following surgery. Full details of the study approval by the Chongqing Medical University Animal Care and ethics committee.

### Intravenous Glucose Tolerance Tests (IVGTT)

Twenty eight ApoE^−/−^ mice fed with HFD were randomly assigned to four groups and treated with different doses of liraglutide (Novo Nordisk, Bagsvaerd, Denmark). The first group (H group, n = 7), received 1 mg/kg, the second group (M group, n = 7), 0.5 mg/kg, the third group (L group, n = 7), 0.1 mg/kg, and the controls (NC group, n = 7), sterile saline. All injections were given intraperitoneally twice daily in a volume of 0.1 ml for 8 weeks. The overnight-fasted mice were given i.v. glucose (1 g/kg body weight) and venous blood was collected before (time 0) and after injection at indicated times for measurement of glucose (Glucometer Elite; Bayer) and insulin. Each blood sample was replaced by the same volume of fresh whole blood from donor mice.

### Hyperinsulinemic–euglycemic Clamp Studies

Hyperinsulinemic-euglycemic clamp studies were performed as described previously [26]. Chronically cannulated, conscious and unrestrained mice were fasted for 8 h before the studies. Insulin (Novo R, 5 mU•kg^−1^•min^−1^) was infused throughout the clamp. Blood glucose was monitored every 10 min in samples obtained from intrajugular venous catheters. 25% Glucose was infused at variable rates to maintain blood glucose at 100 mg/dl. The glucose infusion rate (GIR) and endogenous glucose production rates were calculated as described [26]. Blood samples (50 µl) were collected before the start and at the end of the clamps and used for measurement of plasma glucose, insulin, triglyceride (TG), total cholesterol (TC), low-density lipoprotein cholesterol (LDL-C), high- density lipoprotein cholesterol (HDL-C), and FFA. During the study, each blood sample was replaced by the same volume of fresh whole blood from a donor mouse. At the end of the clamps, mice were anesthetized with a sodium pentobarbital injection. Within 5 min, epididymal white adipose tissue and liver were collected. Each tissue was dissected, frozen immediately using liquid N_2_-cooled aluminum blocks, and stored at −80°C for later analysis.

### Analytical Procedures

For the determination of plasma [3-^3^H] glucose, plasma was deproteinized with ZnSO_4_ and Ba(OH)_2_, dried to remove 3H_2_O, resuspended in water, and counted in scintillation fluid (Ultima Gold; Packard Instrument Co., Meriden, Connecticut, USA). Plasma FFA levels were determined spectrophotometrically using an acyl-CoA oxidase-based colorimetric kit (Wako Pure Chemical Industries, Osaka, Japan). Plasma insulin levels were determined using a commercial insulin enzyme-linked immunosorbent assay kit (Crystal Chem). Plasma glucose was measured using glucose oxidase method. Plasma TG, TC, HDL-C, and LDL-C concentrations were measured using enzymatic colorimetric methods (Sigma, St. Louis, MO). FGF-21 and adiponectin were evaluated using an ELISA kit (Phoenix Pharmaceuticals, Belmont, CA).

### Quantitative Real-time PCR (QRT-PCR)

Total liver or adipose tissue RNA was obtained from frozen tissue (100 mg) using Trizol reagent (Invitrogen, Carlsbad, CA, USA). Purified RNA was used as template for first-strand cDNA synthesis using PrimerScript TM RT reagent Kit (Takara Bio Inc. Otsu, Japan). QRT-PCR was performed with a SYBR Green PCR kit (Takara Bio Inc. Otsu, Japan), and a Corbett Rotor Gene 6000 real-time PCR system (Corbett Research, Sydney, Australia) according to the manufacturer’s instructions. Gene expressions were analyzed using the comparative Ct method and normalized with β-actin. Forward and reverse primers are listed in Table S1.

### Western Blots Analyses

Liver or adipose tissue (60 mg) was homogenized in a lysis buffer (150 mmol/L NaCl, 1% NP-40, 0.5% deoxycholic acid sodium, and 50 mmol/L Tris, pH8.0) and then incubated at 4°C for 30 min before centrifugation (12,000 g at 4°C). Protein levels were measured using a BCA protein quantification kit (Pierce, Rockford, Illinois, USA). One microliter of tissue extracts (70 µg) was separated by SDS-polyacrylamide gel electrophoresis (12% resolving gel) and transferred to PVDF membranes (Millipore) in a transfer buffer containing 20 mM Tris, 150 mM glycine, and 20% methanol. Immunoblots were then blocked in TBS containing 0.1% Tween-20 and 5% skimmedmilk overnight at 4°C and incubated with primary antibodies including anti-adiponectin, anti-FGF-21 (IgG), anti-phospho-FGFR1 or anti-β-Actin (Research Diagnostics Inc.) (1∶500 dilution) for 2 h at room temperature. Following 3 consecutive 5-minute washes in TBST, blots were incubated with HRP-conjugated secondary antibody (Invitrogen) (1∶500 dilution with TBST) for 1 h at room temperature. After 2 washes in TBST and a final wash in TBS, the blots were scanned using the Odyssey Infrared Imaging System (LI-COR Biosciences) and quantification of antigen-antibody complexes was performed using Quantity One analysis software (Bio-Rad).

### Statistical Analysis

Data were presented as means ± SD or SEM. Comparisons among groups were made using ANOVA, followed by a post hoc (PLSD) test to compare two individual groups. Differences were considered statistically significant at *P*<0.05. All analyses were performed using SPSS 13.0 (SPSS graduate pack; SPSS, Chicago, IL).

## Results

### Effects of Liraglutide on IVGTT in HFD-fed Mice

The aim of the IVGTT testing was to identify the most effective dose of liraglutide in HFD-fed ApoE^−/−^ mice. The peak glucose levels achieved during the first 10 min of the IVGTT and were significantly lower in the H group (1 mg/kg) than in the L (0.1 mg/kg), M (0.5 mg/kg) and HF groups. Plasma glucose levels returned to baseline within 30 min in the H group, while the return to baseline was delayed in the M, L and HF groups (Fig. S2A–E). Insulin responses to IV glucose were drastically increased in the H group compared to L and N groups with intermediate values in the M group (Fig. S2F–J). The result indicated that liraglutide 1 mg/kg twice daily produced the most significant glucose changes in HFD-fed mice. Therefore, this dose of liraglutide was used in all subsequent studies.

### Acrp30 Knockdown Aggravates HFD-induced Insulin Resistance in ApoE^−/−^ Mice

**Figure 1:**
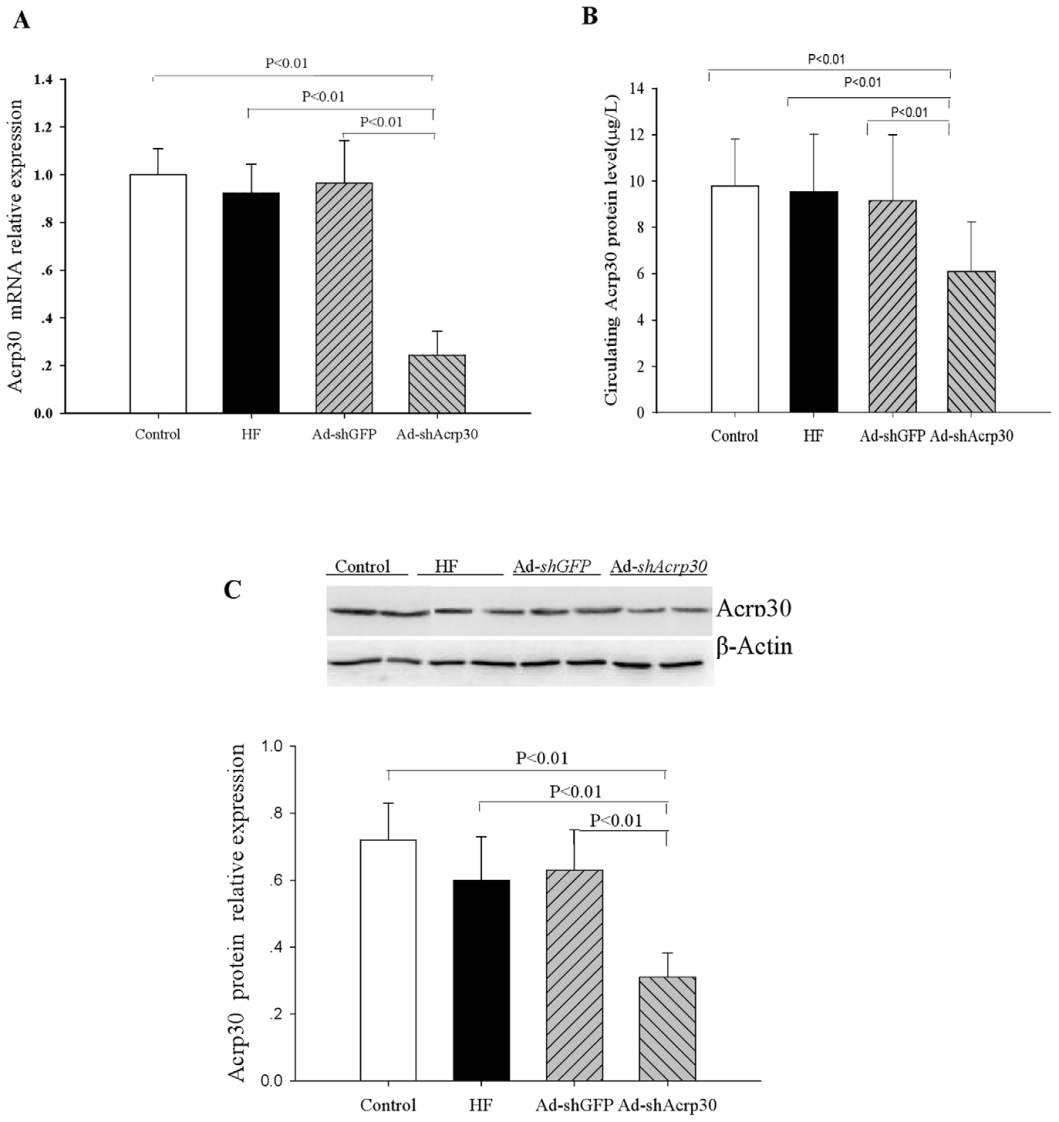
Adiponectin knockdown in HFD-fed ApoE^−/−^ mice (n = 6∼10). Control, mice fed with regular chow; HF, HFD-fed mice treated with saline; Ad-*shGFP*, HFD-fed mice treated with Ad-*shGFP*; Ad-*shAcrp30*, HFD-fed mice treated with Ad-*shAcrp30*. *A*) Relative adiponectin mRNA levels in adipose tissues. *B*) Circulating adiponectin levels. *C*) Adiponectin protein level in adipose tissues.

**Figure 2:**
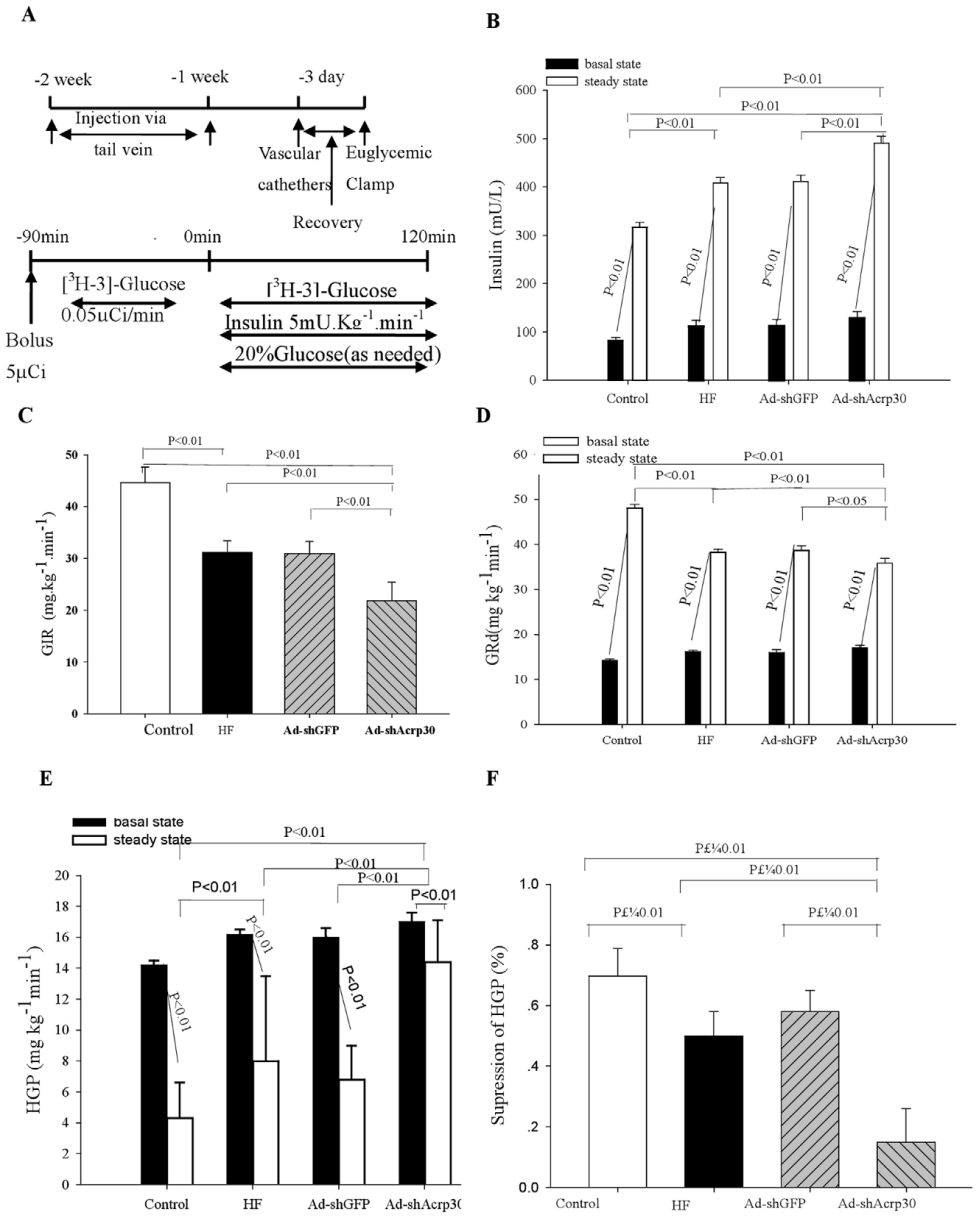
Adiponectin knockdown aggravates HFD-induced insulin resistance. *A*) Experimental design for hyperinsulinemic-euglycemic clamp studies. *B)* Plasma insulin levels before and during euglycaemic– hyperinsulinaemic clamping. *C*) Glucose infusion rate (GIR) during the clamp. *D*) Glucose disposal rate (GRd) before and during the insulin clamp studies. *E*) Hepatic glucose production before and during the clamp studies. *F*) Suppression of glucose production during the clamp period express as percent decrease from basal glucose production. Control, ApoE^−/−^ mice fed with regular chow; HF, HFD-fed ApoE^−/−^ mice treated with saline; Ad-*shGFP*, HFD-fed ApoE^−/−^ mice treated with Ad-*shGFP*; Ad-sh*Acrp30*, HFD-fed ApoE^−/−^ mice treated with Ad-*shAcrp30*. All values are mean ± SE.

Previously validated shRNA sequence against Acrp30 [27] was engineered into an adenovirus vector and given to the HFD-fed ApoE^−/−^ mice. Treatment with Ad-*shAcrp30* achieved a 76% reduction of adiponectin mRNA expression in adipose tissue (Fig. 1A) and a 35% reduction of circulating adiponectin level in the Ad-*shAcrp30* group compared with the control, HF or Ad-*shGFP* groups (6.07±2.1 7 vs. 9.77±2.05, 9.53±2.50 and 9.14±2.87 µg/L, respectively, *P*<0.01, Fig. 1B). The *Acrp30* protein level in adipose tissues was also significantly decreased by the Acrp30 knockdown (a 48.3% reduction as compared with HF group, Fig. 1C). In addition, Ad-*shAcrp30* treatment exhibited clinical signs of glucose and lipid abnormalities and insulin resistance with increased plasma insulin, FFA, TG, TC and LDL-C and decreased HDL-C levels (*P*<0.01 or *P*<0.05, Table 1). To assess the overlapping impacts of HFD, hyperlipidemia, and Acrp30 knockdown on glucose homeostasis and insulin sensitivity, hyperinsulinaemic–euglycaemic clamps were performed after an overnight fast. The experimental design for hyperinsulinemic-euglycemic clamp studies was showed in Fig. 2A. Through Acrp30 knockdown, plasma insulin concentrations were raised to approximately 4 times over basal values in the HFD-fed mice. At identical insulin infusion rates (5 mU kg^−1 ^min^−1^) plasma insulin levels were higher in the Ad-*shAcrp30* group than in the control, HF and Ad-*shGFP* groups (*P*<0.01, Fig. 2B). Despite higher insulin levels, GIR and GRd were significantly lower in the Ad-*shAcrp30* group than in the control, HF and Ad-*shGFP* groups (*P*<0.01, Fig. 2C and D). In addition, insulin’s ability to suppress hepatic glucose production (HGP) during clamps was markedly reduced in the Ad-*shAcrp30* group compared with other groups (*P*<0.01, Fig. 2E and 2F).

**Table 1:** Basal plasma parameters of the experimental groups.

Index	Control (n = 10)	HF (n = 10)	Ad-shGFP (n = 6)	Ad-shAcrp3 (n = 10)
Body weight (g)	27.4±1.2	29.4±1.3▴	28.5±1.2▴	28.9±1.4▴
FBG (mmol/l)	5.9±0.8	7.7±0.8▴	7.7±0.4▴	7.9±0.7▴ *
FFA (mmol/l)	2.21±0.34	3.10±0.0▴	3.10±0.10▴	3.60±0.18▴ *
TG (mmol/l)	1.2±0.2	2.3±0.2▴	2.2±0.2▴	2.7±0.3▴ *
TC (mmol/l)	12.2±3.1	21.9±1.8▴	20.9±2.4▴	24.5±3.1▴ *
HDL-C (mmol/l)	2.5±0.5	4.6±0.8▴	4.5±0.5▴	3.8±0.4▴ *
LDL-C (mmol/l)	6.0±1.7	14.0±2.1▴	13.7±2.2▴	16.9±2.1^▴Δ^
Insulin(mU/L)	82.3±6.3	111.1±13.0▴	112.1±13.6▴	127.9±11.2▴ *

Biochemical parameters represent the average ± SE of at least five basal measurements in each mice.

Control group, regular chow fed ApoE^−/−^ mice; HF, HFD-fed ApoE^−/−^ mice; Ad-*shGFP*, HFD-fed ApoE^−/−^ mice treated with Ad-*shGFP*; Ad-*shAcrp30*, HFD-fed ApoE^−/−^ mice treated with Ad-*shAcrp30*.

▴ *P*<0.01 vs. Control group;

Δ *P*<0.05,

* *P*<0.01 vs. HF group.

### Liraglutide Treatment Attenuated HFD- and Acrp30 Knockdown-induced Insulin Resistance

**Figure 3:**
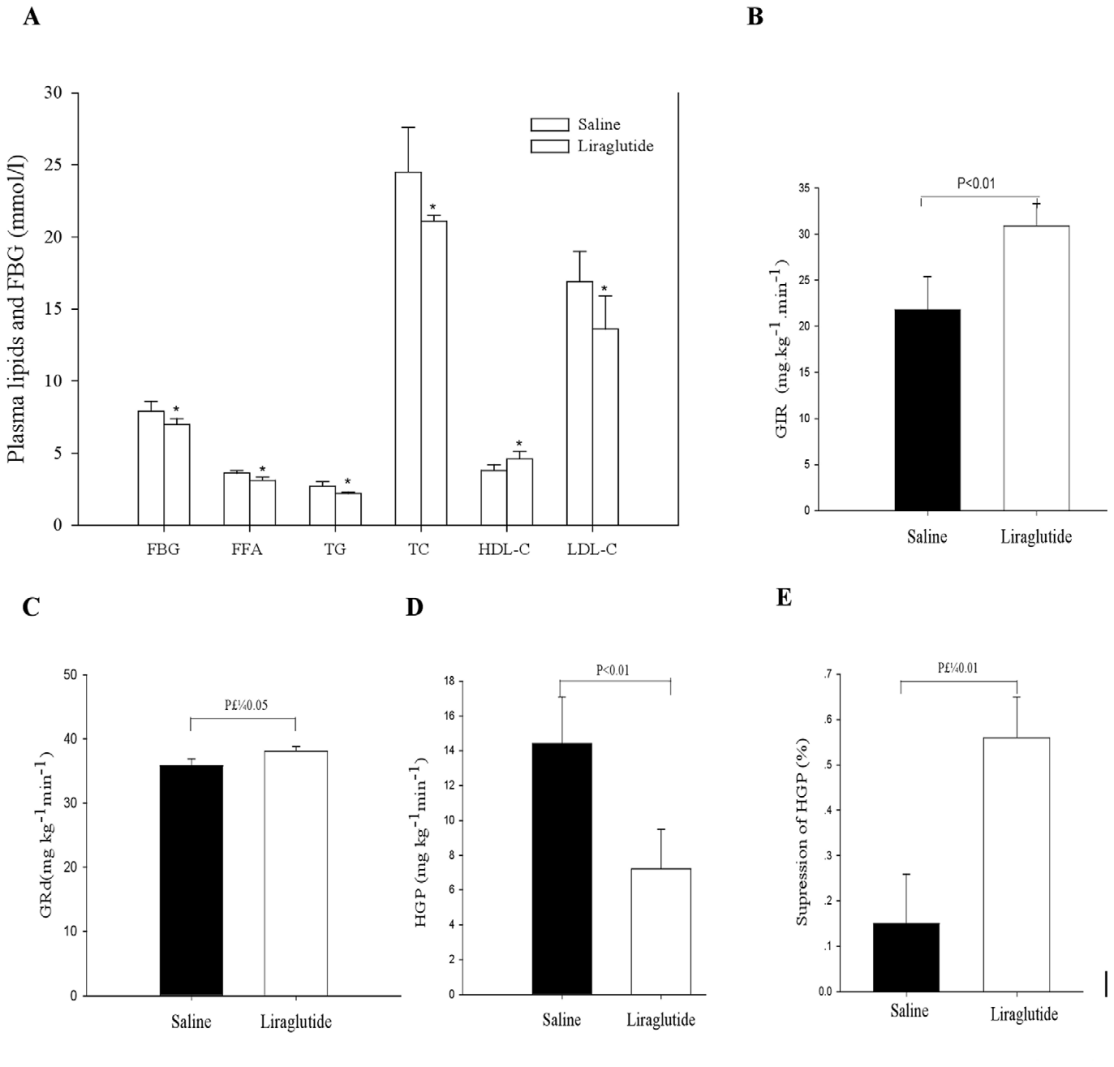
Effects of liraglutide on metabolic parameters and glucose turnover. *A*) Lipids profile and fasting blood glucose (FBG). *B*) Glucose infusion rate (GIR) during the clamp. *C*) Glucose disposal rate (GRd) before and during the insulin clamp studies. *D*) Hepatic glucose production before and during the clamp studies. *E*) Suppression of glucose production during the clamp period express as percent decrease from basal glucose production. All values are means±SE. Saline, HFD-fed ApoE^−/−^ mice treated with Ad-*shAcrp30* plus saline; Liraglutide, HFD-fed ApoE^−/−^ mice treated with Ad-*shAcrp30* plus liraglutide at a dose of 1 mg/kg twice daily. *P<0.05 vs. saline group.

Liraglutide treatment significantly decreased the body weight (26.7±0.9 g vs. 28.9±1.4 g, *P*<0.01) compared with the saline controls. Liraglutide also lowered the increased levels of fasting blood glucose (FBG), FFA TG, TC and LDL-C, and HDL-C in HFD-Acrp30 knockdown mice (All *P*<0.01, Fig. 3A). As shown by hyperinsulinaemic–euglycaemic clamps, liraglutide treatment prevented the decreases in GIR and GRd compared with saline treatment (Both *P*<0.01, Fig. 3B and C). In addition, insulin’s ability to suppress HGP during clamps was also significantly increased by liraglutide treatment (*P*<0.01, Fig. 3D and E).

### Liraglutide Treatment Increases FGF-21 Activity

**Figure 4:**
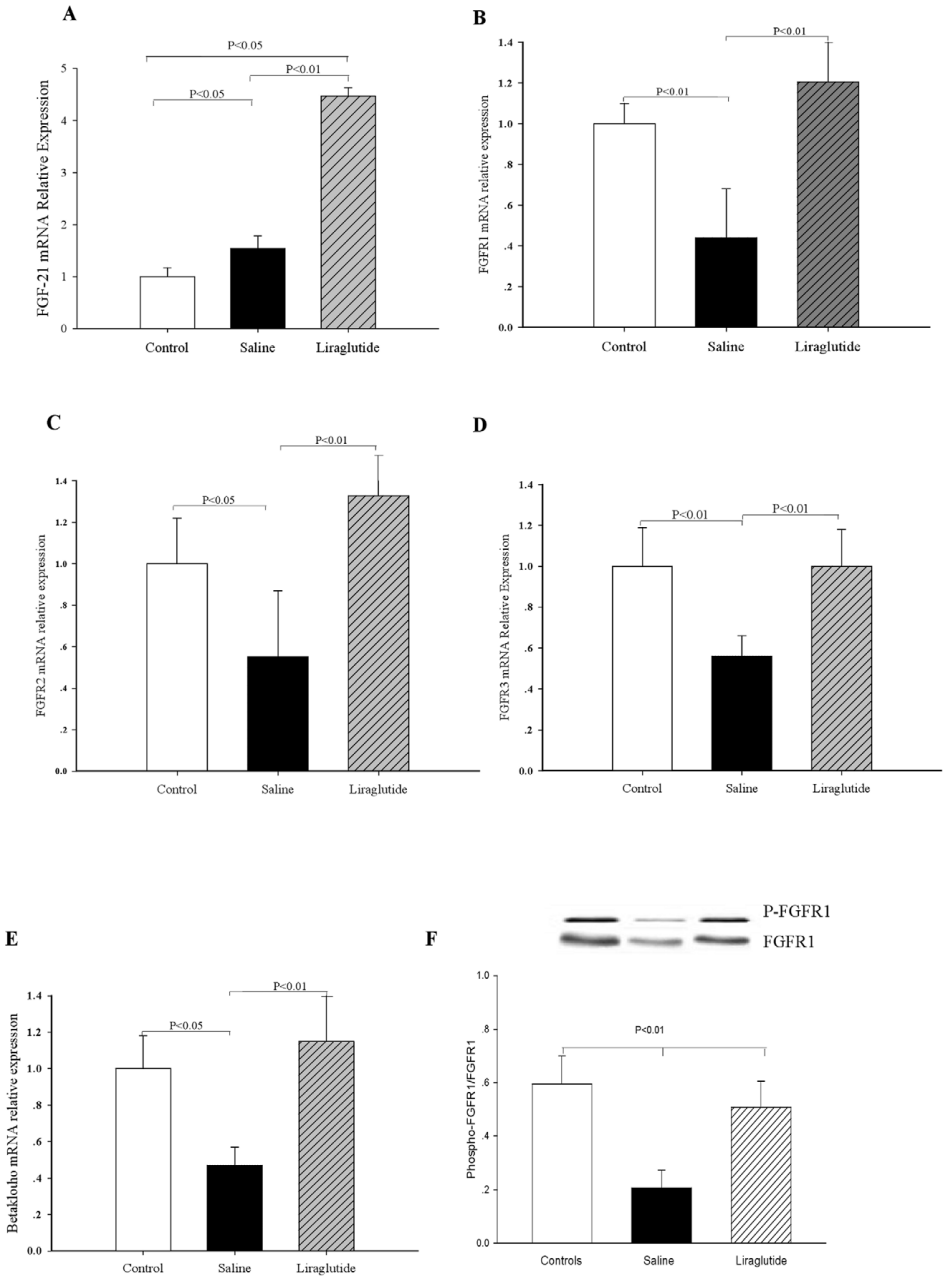
Effects of liraglutide on the FGF-21 and FGF receptors mRNA expression in liver. (A) FGF-21 mRNA expression. (B) FGFR-1 mRNA expression. (C) FGFR-2 mRNA expression. (D) FGFR-3 mRNA expression. (E) β-Klotho mRNA expression. (F) FGFR1 phosphorylation. Controls, ApoE^−/−^ mice fed with regular chow; Saline, HFD-fed ApoE^−/−^ mice treated with Ad-*shAcrp30* and saline; Liraglutide, HFD-fed ApoE^−/−^ mice treated with Ad- *shAcrp30* and liraglutide at a dose of 1 mg/kg twice daily. All values are mean ± SE.

**Figure 5:**
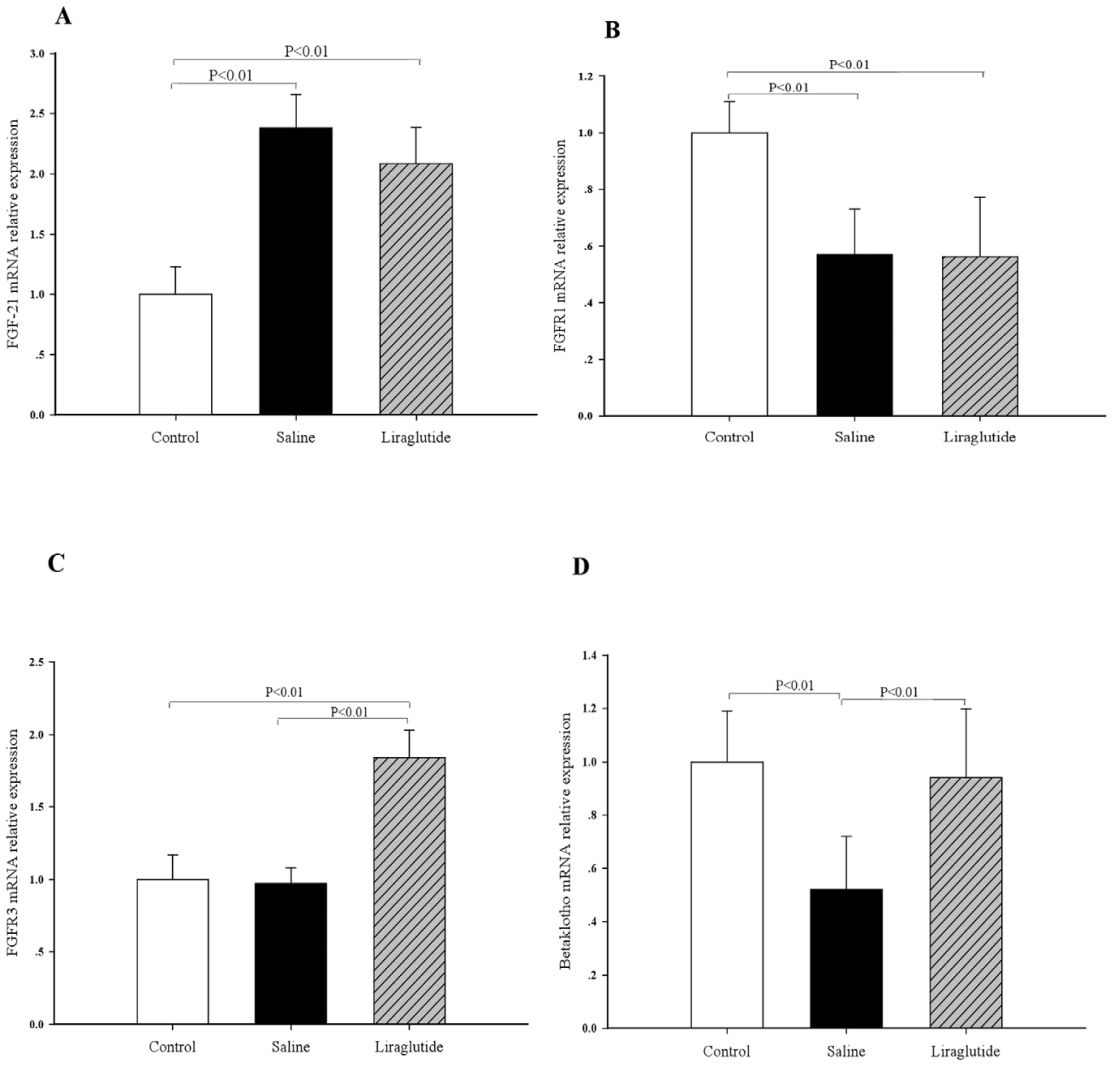
Effects of liraglutide on the FGF-21 and FGF receptors mRNA expression in adipose tissues. (A) FGF-21 mRNA expression. (B) FGFR-1 mRNA expression. (C) FGFR-3 mRNA expression. (D) β-Klotho mRNA expression. Controls, ApoE^−/−^ mice fed with regular chow; Saline, HFD-fed ApoE^−/−^ mice treated with Ad- *shAcrp30* and saline; Liraglutide, HFD-fed ApoE^−/−^ mice treated with Ad-*shAcrp30* and liraglutide at a dose of 1 mg/kg twice daily. All values are means ± SEM.

QRT-PCR analyses revealed that the mRNA expressions of FGF-21 in both liver (∼1.5-fold, *P*<0.05, Fig. 4A) and adipose tissues (∼2.4-fold, *P*<0.01, Fig. 5A) were markedly increased in HFD-Acrp30 knockdown mice compared with controls (regular chow). Liraglutide treatment further up-regulated FGF-21 mRNA expression levels in the liver of HFD- adiponectin knockdown mice (∼2.9-fold, *P*<0.01, Fig. 4A), but not in adipose tissue (Fig. 5A) as compared with saline treatment. In HFD- adiponectin knockdown mice, liver mRNA levels of FGFR-1 were significantly decreased by 55.3% (*P*<0.01, Fig. 4B), FGFR-2 by 45.3%, (*P*<0.05, Fig. 4C), FGFR-3 by 43.6% (*P*<0.01, Fig. 4D) and β-Klotho mRNA by 52.7% (*P*<0.05, Fig. 4E), and adipose mRNA levels of FGFR-1 were decreased by 43.4% (*P*<0.01, Fig. 5B) and β-Klotho by 47.5% (*P*<0.01, Fig. 5D) as compared to controls (regular chow). Liraglutide treatment normalized β-Klotho mRNA (∼41.5%, *P*<0.01, Fig. 5D) and up- regulated FGFR-3 mRNA levels (∼ 87.4%, *P*<0.01, Fig. 5C) in adipose tissue as compared with the saline controls. Treatment of liraglutide also up-regulated liver mRNA levels of FGFR-1 (∼75.8%, *P*<0.01, Fig. 4B), FGFR-2 (∼78.2%, *P*<0.01, Fig. 4C), FGFR-3 (∼43.8%, *P*<0.01, Fig. 4D) and β-Klotho (∼67.6%, *P*<0.01, Fig. 4E). Importantly, liraglutide increased phosphorylation of FGFR1 to the levels observed in ApoE^−/−^ mice fed HFD alone (*P*<0.01, Fig. 4F).

### Liraglutide Treatment Elevates Plasma and Hepatic FGF-21 Protein Levels

**Figure 6:**
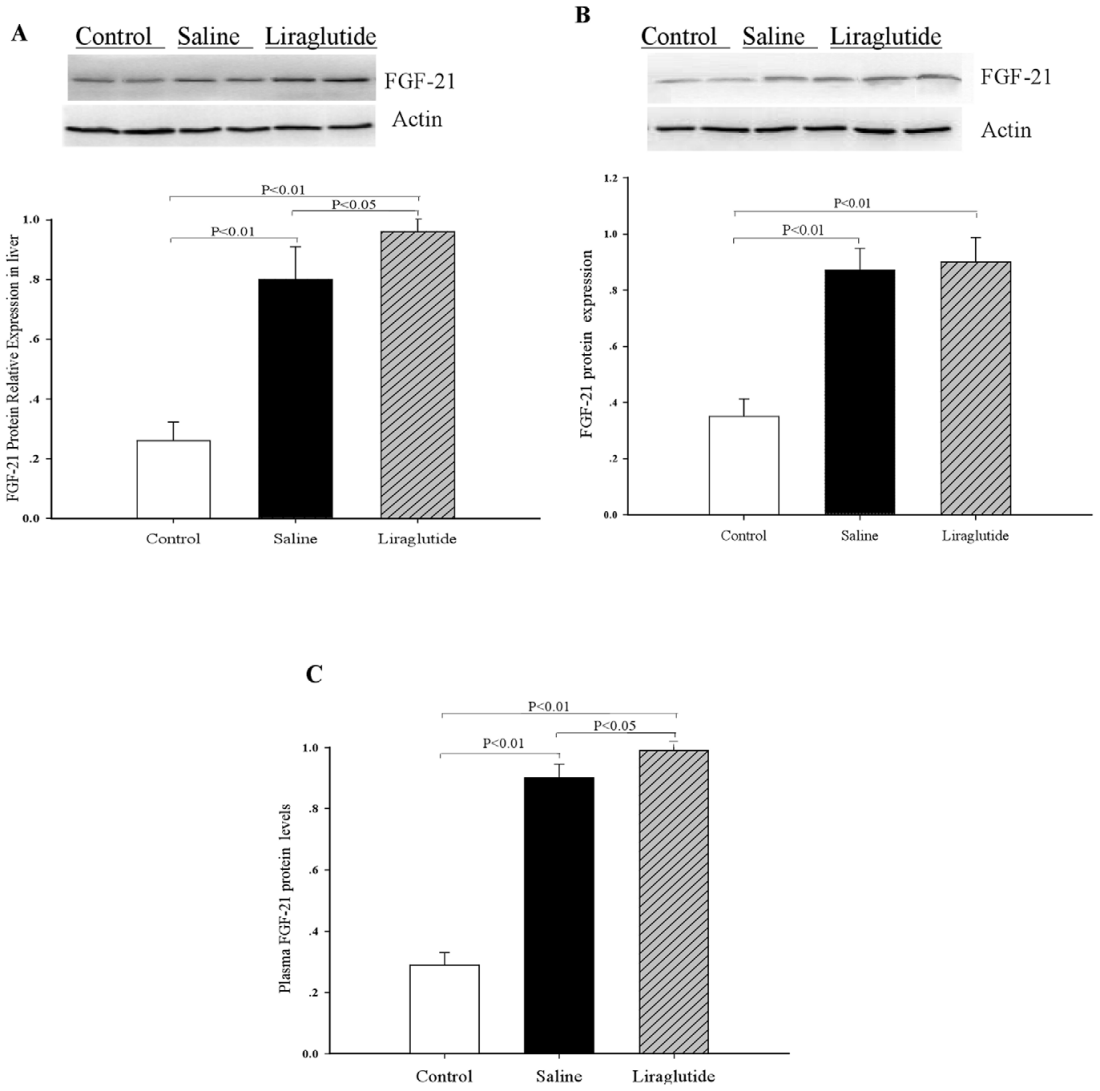
Effects of liraglutide on FGF-21 protein contents. (A) FGF-21 protein level in the liver. (B) FGF-21 protein level in adipose tissues. **(**C) Plasma FGF-21 levels. Controls, ApoE^−/−^ mice fed with regular chow; Saline, HFD-fed ApoE^−/−^ mice treated with Ad-sh*Acrp30* and saline; Liraglutide, HFD-fed ApoE^−/−^ mice treated with Ad-*shAcrp30* and liraglutide at a dose of 1 mg/kg twice daily. All values are means±SE.

Western blot analyses revealed a marked increase in FGF-21 protein levels in liver (∼3.1-fold, *P*<0.01, Fig. 6A), adipose tissue (∼2.5-fold, *P*<0.01, Fig. 6B), and plasma (∼3.1-fold, *P*<0.01, Fig. 6C) in HFD- adiponectin knockdown mice compared with the controls (regular chow). Liraglutide treatment further increased FGF-21 protein levels in liver (∼1.2-fold, *P*<0.01, Fig. 6A) and plasma (∼1.1-fold, *P*<0.01, Fig. 6C) compared with saline controls.

## Discussion

New findings of this work were the changes of FGF-21 and its receptors in a new animal model where insulin resistance was induced by the combination of HFD, ApoE deficiency and adiponectin knockdown. Liraglutide increased FGF-21 and FGF receptor mRNA and protein contents and improved insulin action in this mouse model. This may add a new mechanism for liraglutide induced improvement of insulin sensitivity.

T2DM is commonly associated with insulin resistance, dyslipidemia and hypoadiponectinemia. To simulate the human conditions as closely as possible, we have created a mouse model where IR was produced by feeding a HFD to ApoE^−/−^ mice which are dyslipidemic [23] and were made hypoadiponectinemic with Ad-sh *Acrp*30. Here, the major advantage of using shRNA is to decrease specific gene expression in a normal adult animal and to avoid the confounding compensatory developmental affects often associated with gene knockout mouse models. In this new insulin resistance model, adiponectin mRNA expression was reduced by 76% in adipose tissue, and plasma Acrp30 level was reduced by 35%, showing that HFD plus adiponectin knockdown further reduced adiponectin transcription and release compared with mice fed with HFD alone. These results may also reflect differential effects of Ad-sh*Acrp30* on adipose and other tissues because adiponectin was secreted not only by adipose tissues. Importantly, during hyperinsulinemic- euglycemic clamp, HFD- adiponectin knockdown mice showed further increased HGP and decreased GRd compared with mice fed with HFD alone, indicating that HFD feeding and hypoadiponectinemia had an additive effect on insulin resistance in vivo.

Acrp30/adiponectin is known to improve insulin resistance [28] and FGF-21 also enhances insulin sensitivity in animals and humans with diabetes [29], [30]. In the present study, the mRNA expression of FGF-21 in liver and adipose tissue was significantly up-regulated in insulin resistance mice induced by HFD-adiponectin knockdown. This increase was paralleled by an increase FGF-21 protein in liver, adipose tissue and plasma. These results were consistent with those we have observed in T2DM patients [15]. We speculate that increased FGF-21 contents in liver and adipose tissues may be a compensatory mechanism to reduce insulin resistance.

To further observe the changes of FGF-21 and its receptor action, we examined the mRNA expression of key components involving FGF-21 signaling pathway, including FGF receptor 1–4 (FGFR1-4), β-Klotho and phosphorylation of FGFR1. FGF-21 initiates its signaling cascade by binding to the FGF receptor which has 4 isoforms, FGFR-1, 2, 3 and 4. β-Klotho is necessary for FGF-21 binding to FGFRs. Cells lacking β-Klotho do not respond to FGF-21, and the introduction of β-Klotho to these cells confers FGF-21-responsiveness [30]. In the present study, we found that Acrp30 knockdown further decreased FGFR-1 and β-Klotho mRNA levels in adipose tissue as well as FGFR-1, FGFR-2, FGFR-3, β-Klotho mRNA levels and phosphorylation of FGFR1 in the liver in HFD-fed mice, suggesting that FGF-21 signaling pathway was inhibited. Interestingly, despite high endogenous FGF-21 levels in obese mice and T2DM patients, exogenous FGF21 administered at pharmacologic doses appears to exert actions to improve metabolic parameters and induces weight loss [31], [32]. A state in which high endogenous levels of a physiologic regulator appear to be ineffective but in which high pharmacologic doses induce the expected results suggests a state of hormone resistance. Similarly, obesity has been identified as a state of leptin resistance in which high circulating levels fail to induce the desired physiologic effect of decreased feeding [33]. Thus, it seems likely that there is a state of FGF-21 resistance in obesity or diabetes. As we demonstrate in this article, the up-regulation of FGF-21 together with the down-regulating of FGFR, and β-Klotho in liver and adipose tissues in this novel model further support the concept of FGF-21 resistance through a different approach.

Our previous findings indicated that exenatide, a GLP-1 analogue, prevented fat-induced insulin resistance [13]. In the current study, we investigated the impact of liraglutide on insulin resistance induced by the combination of HFD, ApoE deficiency and adiponectin knockdown in vivo. Except for its known effects on body weight, fasting blood glucose and lipid, liraglutide restored adiponectin expression to levels observed in ApoE^−/−^ mice fed HFD alone (data not shown), dramatically elevated FGFR-3 mRNA levels and restored β-Klotho mRNA expression in adipose tissue. It also restored mRNA levels of FGFR-1-3, β-Klotho mRNA expressions and effectively increased phosphorylation of FGFR1 in liver. These data suggested that liraglutide might tissue specifically activate FGF-21 transcriptional activity in adipose tissue through the β-Klotho-FGFR-3 complex and in liver through β-Klotho-FGFR-1-3 complex. In parallel with increased β-Klotho-FGFR activation, liraglutide also slightly increased FGF-21 mRNA and protein expression in the liver as well as FGF-21 plasma levels in HFD- adiponectin knockdown animal. Thus, it seems likely that increased β-Klotho-FGFR and FGF-21 activation induced by liraglutide to achieve a pharmacologic levels lead to the improvement of FGF-21 resistance, and accompanying increased insulin sensitivity. Based on our present findings, it is likely that liraglutide may have a direct effect on FGF-21 activity.

In conclusion, our work provided a compelling insulin resistance model, in which the combination of HFD and adiponectin knockdown exacerbated insulin resistance and probably also resulted in FGF-21 resistance. Liraglutide increased adiponectin levels and FGF-21 activity and was sufficient to prevent severe insulin resistance induced by adiponectin knockdown, and thereby also probably improved FGF-21 resistance. These results advanced the notion that increasing FGF-21 activity may be a novel strategy to the treatment of insulin resistance or diabetes.

## Supporting Information

Figure S1 Ad-sh*Acrp30* suppressed adiponectin mRNA expression in a time- and dose-dependent manner in adipose tissues. (A) Dose response for adiponectin mRNA expression after Ad-sh*Acrp30* treatment. (B) Time course of effects of Ad-sh*Acrp30* (100 µl, 1×10^9^ PFU) on adiponectin mRNA. The average values ± SE of three independent experiments are shown.
(TIF)

Figure S2 Intravenous glucose tolerance test (IVGTT) (n = 7). (A–E) Glucose curves in four groups. (F–J) Insulin curves in four groups. Values are presented as means ± SE, *P<0.05, ^#^ *P*<0.01.
(TIF)

Table S1 Characteristics of the primers used for RT-PCR analysis.
(DOC)
