# Supplementary figures and images for: Liraglutide Increases FGF-21 Activity and Insulin Sensitivity in High Fat Diet and Adiponectin Knockdown Induced Insulin Resistance
Source: PLoS One. 2012 Nov 12;7(11):e48392. doi: 10.1371/journal.pone.0048392 (PMC3495944; doi:10.1371/journal.pone.0048392)

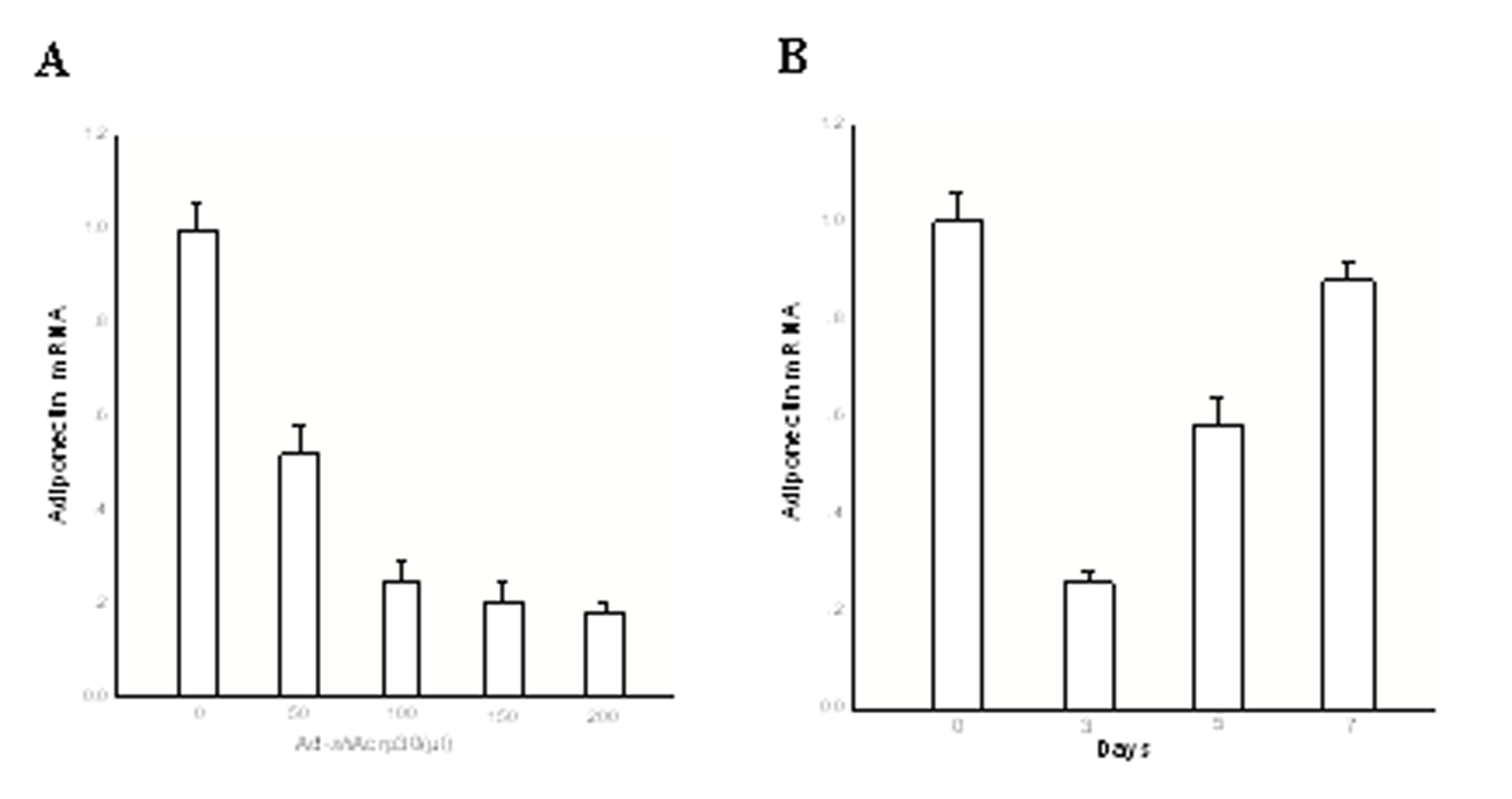

Supplement: Figure S1 — Ad-shAcrp30 suppressed adiponectin mRNA expression in a time- and dose-dependent manner in adipose tissues. (A) Dose response for adiponectin mRNA expression after Ad-shAcrp30 treatment. (B) Time course of effects of Ad-shAcrp30 (100 µl, 1×10^9^ PFU) on adiponectin mRNA. The average values ± SE of three independent experiments are shown. (TIF) [file pone.0048392.s001.tif]

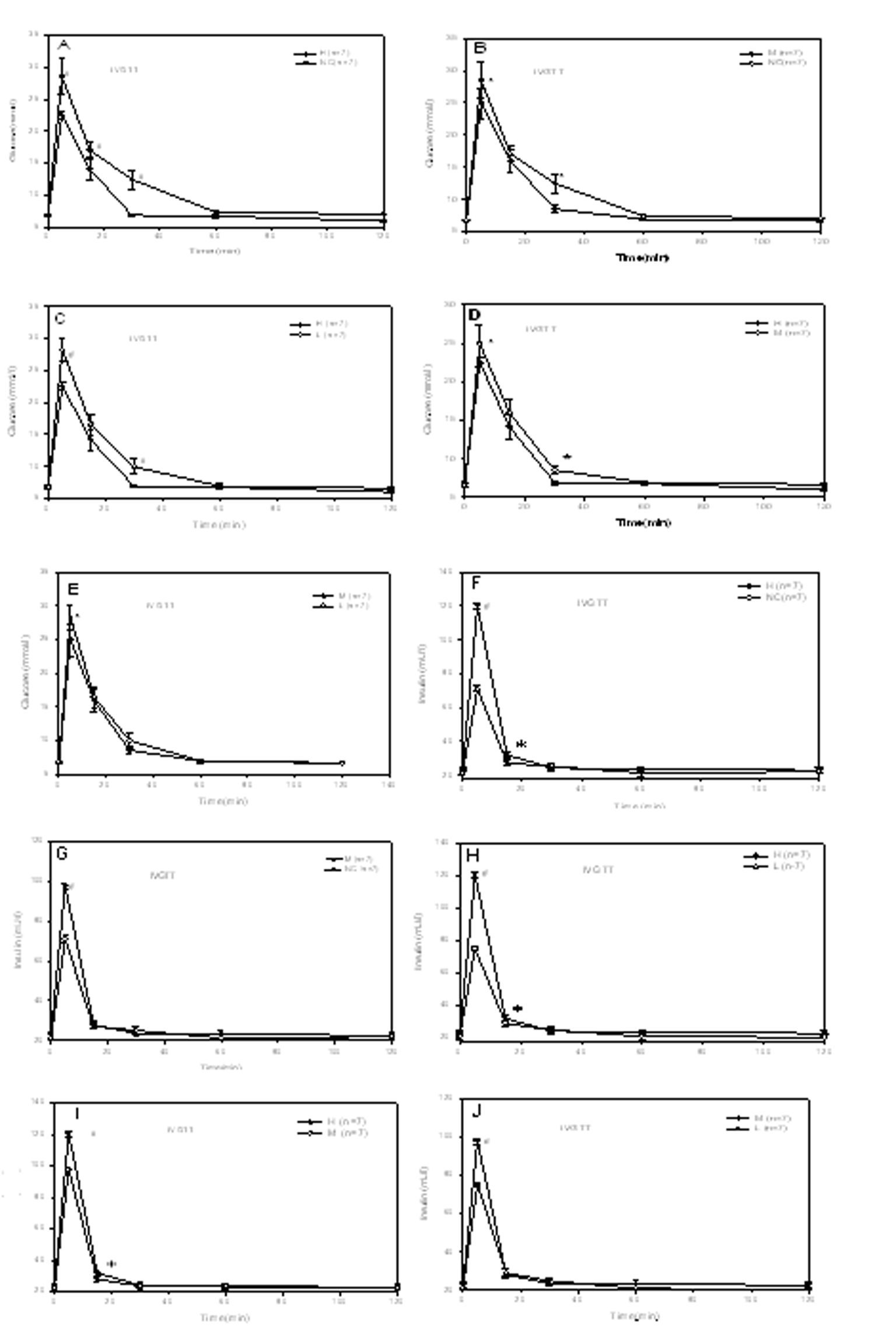

Supplement: Figure S2 — Intravenous glucose tolerance test (IVGTT) (n = 7). (A–E) Glucose curves in four groups. (F–J) Insulin curves in four groups. Values are presented as means ± SE, *P<0.05, # P<0.01. (TIF) [file pone.0048392.s002.tif]
