# Supplementary material for: Liraglutide Increases FGF-21 Activity and Insulin Sensitivity in High Fat Diet and Adiponectin Knockdown Induced Insulin Resistance
Source: PLoS One. 2012 Nov 12;7(11):e48392. doi: 10.1371/journal.pone.0048392 (PMC3495944; doi:10.1371/journal.pone.0048392)
Supplement: Table S1 — Characteristics of the primers used for RT-PCR analysis. (DOC) [file pone.0048392.s003.doc]

**Table S1** Characteristics of the primers used for RT-PCR analysis

| Gene | Forward and reverse primers | Amplified fragment (bp) |
| --- | --- | --- |
| β-actin | 5’-CCACTGCCGCATCCTCTTCCTC-3’ | 400 |
|  | 5’-TCCTGCTTGCTGATCCACATCT-3’ |  |
| Acrp30 | 5’-CTCTTAATCCTGCCCAGTCAT-3’ | 507 |
|  | 5’-GAGGCTCACCTTCACATCTTT-3’ |  |
| FGF-21 | 5'-CACCGCAGTCCAGAAAGT-3' | 385 |
|  | 5'-CCTGTAAAGGCTCTACCATG-3' |  |
| betaklotho | 5’-TGGGGTCCCATTGGATAGAG-3’ | 129 |
|  | 5’-ACTCAGGGTAGTCGCCGTC-3’ |  |
| FGFR-1 | 5’-TGTTTGACCGGATCTACACACA-3’ | 62 |
|  | 5’-CTCCCACAAGAGCACTCCAA-3’ |  |
| FGFR-2 | 5’-TCGCATTGGAGGCTATAAGG-3’ | 64 |
|  | 5’-CGGGACCACACTTTCCATAA-3’ |  |
| FGFR-3 | 5’-GCATCCTCACTGTGACATCAAC-3’ | 70 |
|  | 5’-CCTGGCGAGTACTGCTCAAA-3’ |  |
| FGFR-4 | 5’-CGCCAGCCTGTCACTATACAAA-3’ | 77 |
|  | 5’-CCAGAGGACCTCGACTCCAA-3’ |  |
| cFos | 5’-AGGCAGAACCCTTTGATG-3’  5’-CAGGTGACCACGGGAGTA-3’ | 196 |

FGF-21, Fibroblast growth factor-21; FGFR, FGF receptor.
